# A method to detect immunoreactions on the basis of current *vs.* concentration slope – an electrochemical approach†

**DOI:** 10.1039/d0ra07582e

**Published:** 2020-12-18

**Authors:** Jagriti Gupta, Jyoti Bisht, Mini Agrawal, Jaydeep Bhattacharya, Prasenjit Sen, Ranjita Ghosh Moulick

**Affiliations:** School of Biotechnology, Jawaharlal Nehru University New Delhi 110067 India; Department of Physics and Astronomy, NIT Rourkella Odissa 769008 India; Amity Institute of Integrative Sciences and Health, Amity University Haryana, Amity Education Valley Haryana – 122413 India ranjita.ghoshmoulick@gmail.com; School of Physical Sciences, Jawaharlal Nehru University New Delhi 110067 India

## Abstract

The emergence of novel infectious diseases is rising with time and is a major threat to the society. The recent outbreak of infectious coronavirus disease has made a huge impact in our lives. The massive outbreak of the disease revealed that there is room for development of new diagnostics tools and methods to screen huge numbers of samples in the shortest possible time. Our current work relates to an electronic diagnostic system and method that rapidly detects the presence of an antigen in solution. Our designed system is capable of separating the immunocomplex formation on the basis of the slope it produces in contrast to the controls, when oxidation peak current is plotted against the concentration of the reactant after electrochemistry measurement. In this system, antibody conjugated copper nanoparticles synthesized by the electroexplosion method has played the key role. The values of the slopes of copper nanoparticles (CuNPs) was found to be −3.7637, whereas those for CuNP conjugated Antibody and CuNPAntibodyAntigen were −2.3044 and −0.8332, respectively. Hence, the current method could become one of the easiest and fastest method for the electronic detection of an immune reaction and a good replacement for the time-consuming, label-based assays in multistep reaction.

## Abstract 2

Synthesis of Cu nanoparticle by Electro-Explosion of Wire (EEW) technique for immunosensing application.

## Graphical abstract

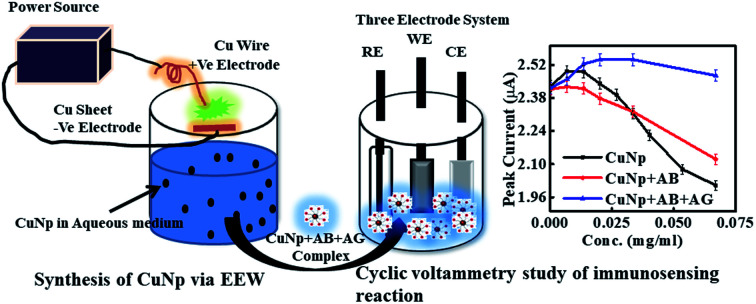

## I. Introduction

Emerging infectious diseases (EIDs) cause significant public health consequences and burden on the economy. Several complex factors for *e.g.* environmental or ecological change, socio-economic background, population pressure, genetic variation *etc.* trigger emergence and wide spread of these diseases. Emergence or re-emergence of such diseases is continuously rising over time.^1,2^ A long list of infectious disease can be seen on the WHO's website (https://www.who.int/topics/infectious_diseases/en/). Leprosy, Leishmaniasis, Malaria, Tuberculosis, Dengue fever, Poliomyelitis, influenza, cholera, small pox *etc.* are a few to be named. Currently, the outbreak of novel corona virus (SARS COV-2) has taken more than 1 600 000 lives worldwide. Its high infectivity resulted in a large number of infected individuals and death. A great deal of emphasis is given on the research and development of techniques for the diagnosis of infectious diseases in the laboratories of developing countries. Different diagnostics approaches such as assessment through signs and symptoms, simple culture techniques, staining and examining under the microscope, radiology, molecular biology and serologic techniques are used by the health care system. But the real challenge demands massive testing and re-testing of the samples in the shortest span of time.^3^ While the frontline tests, RTPCR or immunoassays^4^ are widely used and are reliable diagnostics methods, the major drawback of these processes are the analysis time, which is very lengthy. Point-of-care biosensor devices have the potentials to serve as the rapid testing devices so that the best possible measures could be taken. There are developments of much faster and simpler biosensor devices based on chip, paper or other material (LAMP PCR, test strip to detect IgG/IgM, graphene based electrodes) that offer quick decision making over conventional techniques.^5,6^

In our current work, we generated bare copper nanoparticles (Cu NP) by electro-explosion of wire (EEW) technique.^7^ This technology is a promising method for nanopowder preparation and is based on the explosion of a thin conducting metal wire triggered by a large current density pulse passed through that wire. Cu NP prepared through this technique had been well-studied^8–10^ and is known for its high electrical conductivity, high melting point and low-cost usage.^11^ Easy availability of Cu wire and Cu plate and possibility of large scale synthesis encouraged us to work with these particles. The particles produced by EEW technique were tried for various applications. Like, development of contacts in the area of photovoltaics or microelectronics^12^ and in printed electronic circuits.^13^ New strategies were also reported in order to achieve high electrical conductivity^14^ or in case of composite synthesis.^15^ These particles had also been tested in the field of biology for their antimicrobial activity.^16,17^ In an earlier publication we have described about the distinctive interaction of hemoglobin with CuNP prepared through the EEW method.^18^ But in our current approach, the synthesized Cu nanoparticles (colloidal solution) were conjugated to the antibodies and immunoreaction was conducted between the antibody coated CuNP with its respective antigen. The measurement was done using electrochemistry (cyclic voltammetry). The oxidation/reduction current with increase in reactant concentration was measured separately for CuNP, antibody conjugated CuNP and antibody–antigen conjugated CuNP. When the immune reaction was plotted with oxidation current and concentration, it generated a variable slope from the control reactions that reflected the presence of the analyte (infection). The slope values are found in the range of (a) CuNP = −3.7637; (b) CuNP conjugated Antibody = −2.3044; (c) CuAntibodyAntigen = −0.8332.

## II. Experimental

### A. CuNP preparation by EEW method

**Fig. 1:**
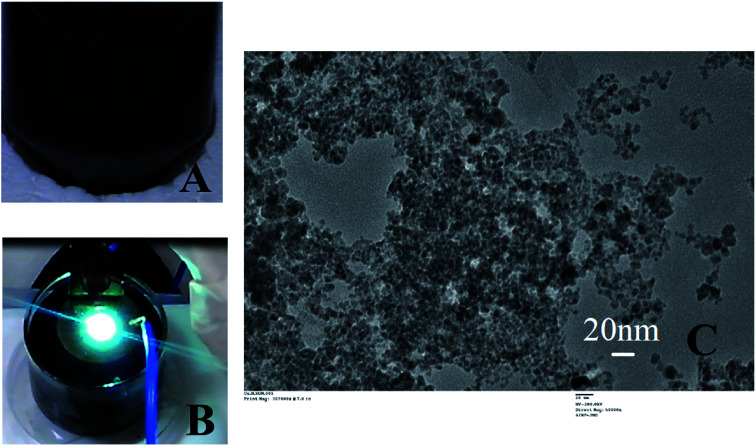
Summarizes the CuNP preparation and characterization. The CuNP particles just after preparation is shown in (A). (B) shows the reaction vessel with a single explosion with characteristic green flame. The TEM image of these particle is shown in (C).

The entire preparation of nanoparticles through the wire explosion [7] was carried out using a Cu wire (0.5 micron in diameter, 99.9% pure) and a Cu plate (99.9% purity) ESI Fig. 1,† immersed in 0.2 micron filtered MilliQ water (medium). The wire was driven through a wire guide and exploded onto the Cu plate (Fig. 1). DC voltage power supply (Billionix) was used to pass instantaneous currents of 50 A, applied at a voltage of 36 V to get the desired size of copper nano particles. After explosion the particles were retained suspended in the same medium till further processing. In order to achieve explosion from the copper wire of length 100 cm, a 100 ml reaction vessel was prepared. After preparation, the particles were collected in falcon tubes and the supernatant was collected. The stock concentration of the CuNP was 0.134 mg ml^−1^.

### B. Antibody conjugation and characterization

Anti GFP polyclonal antibody from Thermo Fischer Scientific and GFP Recombinant Aequorea Victoria Protein from Invitrogen have been used for conjugation study. 10 μg ml^−1^ anti GFP antibody and 0.0134 mg ml^−1^ CuNP was used for coating. The antibody was mixed well with the CuNP, kept at 4 °C for a day, washed 3 times by centrifugation to get rid of the unbound antibody.

#### 1. ZETA and DLS

Malvern Zetasizer NanoZS was used for the zeta potential and DLS measurement. 1 ml of sample was used for measurement keeping the CuNP and the antibody concentration in the above-mentioned ratio.

#### 2. Fluorescence microscopy

The antibody coated CuNP was incubated with 10 μg ml^−1^ GFP Recombinant Aequorea Victoria Protein for 1 hour in dark then centrifuged at 3000 rpm for 10 min. Pellet was collected and drop casted on a clean glassslide. The samples were checked with Olympus Flu view FV1000 for Green fluorescence from the GFB bound samples.

#### 3. TEM and XRD

The CuNP prepared in water was monitored through transmission electron microscopy, TEM (JEOL 2100F, Japan) and X-ray diffractometer (PANalytical 2550-PC X-ray diffractometer) before and after antibody conjugation. The samples were prepared by collecting the pellet after centrifugation to achieve high concentration and drop casting the same on TEM grids.

### C. Cyclic voltammetry study

The electrochemical measurements were performed *via* PGSTAT204 electrochemical workstation (75 W 50/60 Hz made in Netherlands) with conventional three electrode system with a NOVA 1.11 software. Indium tin oxide coated glass electrode (5 mm × 5 mm) with resistivity 10–15 ohm cm were used as working electrode, a platinum sheet (5 mm × 5 mm) was used as counter electrode and an Ag/AgCl electrode purchased from BASi used as reference electrode. All measurements were done using PBS buffer (0.1 M, pH-7) with KCl (0.01 M) as supporting electrolyte and K_3_FeCN_6_/K_4_FeCN_6_ (0.3 M) as a redox solution. This solution enables easy electron transport over the electroactive species.

## III. Result and discussion

Exploding wire is a phenomenon that has widely been employed by the physicists to study plasma but currently is being utilized for large scale production of highly pure, monodispersed and chemically active nanoparticles. Parameters such as material type, wire dimensions, pulse current, voltage and medium in which the process is carried out can control the size of the nanoparticles. The freshly prepared colloidal solution of CuNP were found to be black in colour as shown in Fig. 1A. In Fig. 1B an image of the reaction vessel (filled with water) employed during the particle preparation with the characteristic bright green flame is shown. The generation of an underwater electric arc in between the two electrodes results in a strong explosion. The explosion is triggered by the electrodynamic force having high pulse current amplitudes (a few hundred amperes), which create an imbalance between the electromotive force and ohmic potential in the wire and result in the splitting of the wire into several segments.^7^ The particles, those were prepared through this method were very small in size having the diameter in the range of 5–20 nm measured by TEM (ESI Fig. 2†). The X-ray diffraction pattern revealed presence of metallic copper indexed by diffraction peak at 2*θ* = 43.28 (ESI Fig. 3†).

**Fig. 2:**
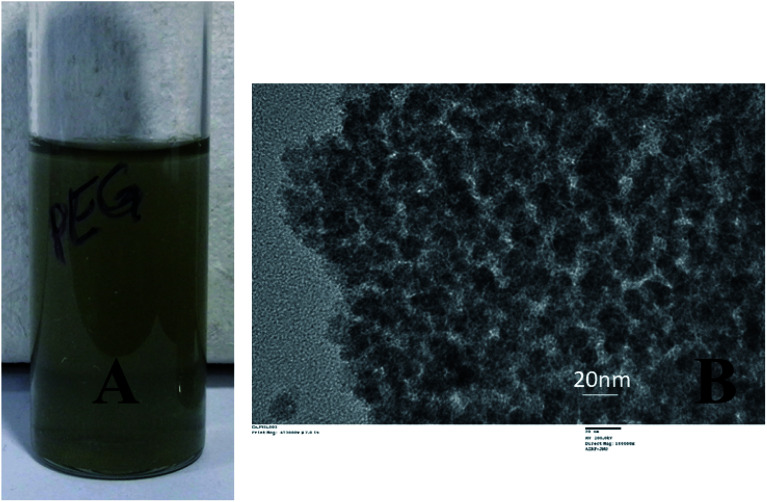
Exhibits PEG coated particles in solution (A) and the TEM image of the same (B).

The CuNP collected from the respective solvent (water) was found to be very active and when mixed with PEG-400, they rapidly bind to it through surface adsorption technique. A dark green colloidal solution (Fig. 2A) indicated formation of PEG (polyethylene glycol) coated particles when 0.125 M PEG-400 were mixed to the solution. The particle remained suspended in solution even after months and never tend to aggregate. PEG acted as a stabilizer and prevented particles from aggregation.^19–21^ The TEM image of the pegylated particle is shown in Fig. 2B which is different from the TEM image of the bare particle (Fig. 1C). Nanoparticles synthesized by the chemical methods in the laboratories, requires capping agents for stability. And the surfaces of the particles are covered with the functional groups of those agents. Therefore, attachment of linker or surface chemistry steps are necessary to bind a molecule to those nanoparticles. But in this EEW method, no stabilizer is needed and bare metallic nanoparticles could be synthesized. The interaction between bare metallic nanoparticles with the respective binding molecule happens instantly.

**Fig. 3:**
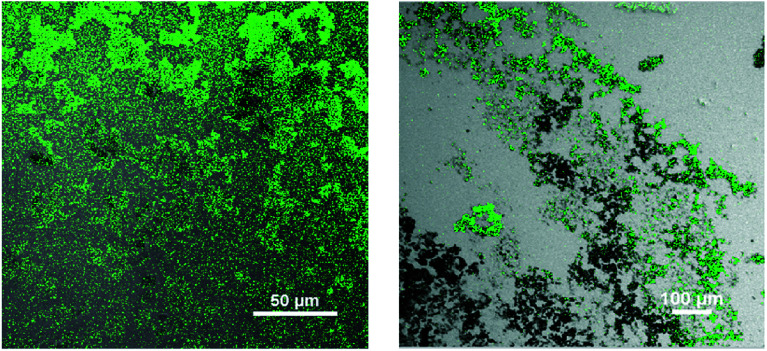
Fluorescence image of anti GFP antibody conjugated CuNP and then bound to GFP (GFP stands for Green Fluorescence Protein).

After successful coating with PEG, the CuNP were conjugated with an anti GFP antibody using the same surface adsorption technique. When antibodies were added to the newly synthesized particles, physisorption occurred. In short, the particles were mixed with anti GFP antibodies on Day 0 (denotes day of synthesis of the nanoparticle) and stored at 4 °C. After a day the particles were washed to remove the unbound proteins. To confirm the antibody conjugation, GFP was reacted to the anti GFP antibody conjugated Cu particle and fluorescence image was captured under the microscope (shown in Fig. 3). The green fluorescence in the image confirmed antibody conjugation to the nanoparticle. Therefore, without any linker molecules, antibodies could be attached to these metal particles with proper orientation.

**Fig. 4:**
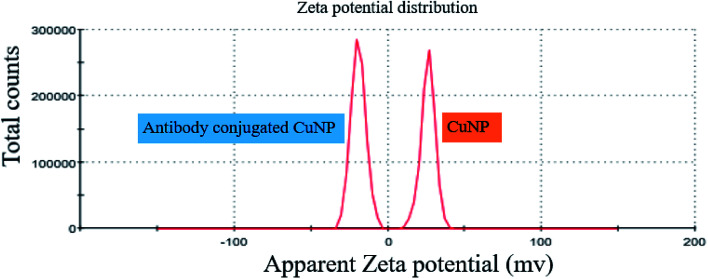
Exhibits the zeta potential values of CuNP prepared in water and after conjugation with anti GFP antibody.

The binding event of anti GFP antibody to the CuNP, was monitored through zeta potential measurement, which showed that the zeta potential value for the bare CuNP (*i.e.* before conjugation) to be +26 mV. On mixing with antibody, the zeta value rapidly shifted to −19 mV and the value remained fixed. This happened instantly on mixing both the solutions. The recording of the zeta value withdrawn from the mixer at a time interval of 3 min is shown in the ESI Table 1,† which did not show any change. The simultaneous size measurement (DLS) experiment showed that the hydrated NP and hydrated protein molecules when mixed together formed complexes of smaller size, which was found to be almost static (ESI Fig. 4†). The study showed that the conjugation process was a fast reaction (Fig. 4).

**Fig. 5:**
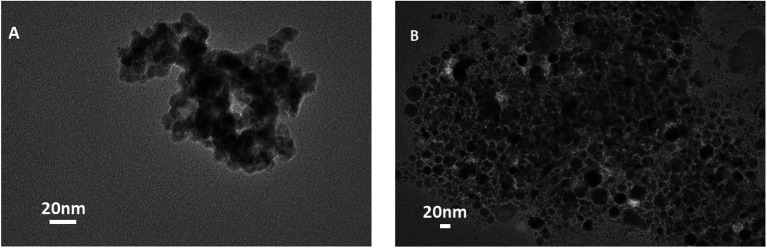
(A and B) Show TEM images of CuNP after antibody conjugation.

The particles after antibody conjugation were also characterized by TEM (Fig. 5). The measurement revealed that a significant change in particle arrangement is observed after bioconjugation. We couldn't see any noticeable difference in the XRD result (data not shown) of CuNP after conjugation with antibody, which proved that bioconjugation did not change the crystal structure of the particles and it was only a surface property.

**Fig. 6:**
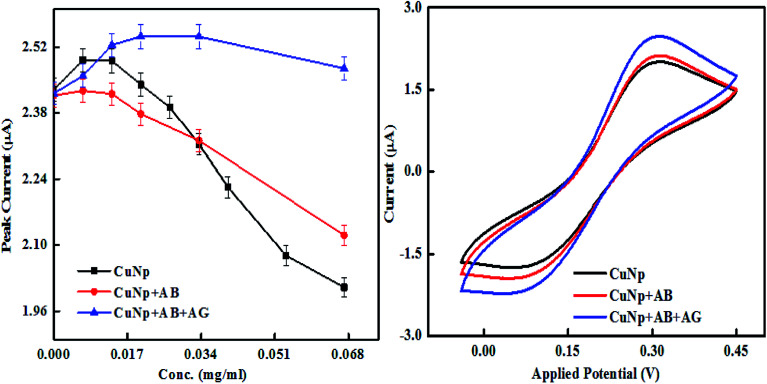
Cyclic voltammetry study of the immunosensing reaction, the peak current *vs.* concentration is shown together with current *vs.* potential curve at 0.067 mg ml^−1^.

We further characterized the particles, electrochemically, through cyclic voltammetry measurements. The experiment was done to find out the differences in the oxidation and reduction potential of the metal nanoparticles prepared by EEW technique before and after bioconjugation and after immunoreaction. Cyclic voltammetry was studied in a PBS buffer (0.1 M, pH-7) with KCl (0.01 M) as supporting electrolyte and K_3_FeCN_6_/K_4_FeCN_6_ (0.3 M) as a redox solution. ITO coated glass electrode (5 mm × 5 mm) with resistivity 10–15 ohm cm is used as a working electrode, platinum sheet and Ag/AgCl as counter and reference electrodes respectively. When the concentration of bare CuNP, CuNP–antibody conjugates (CuNP + AB) and CuNP–antibody–GFP complex (CuNP + AB + AG) is plotted against the peak current (oxidation), a significant difference was observed as illustrated in Fig. 6. The potential *vs.* peak current graph at 0.067 mg ml^−1^ is shown on the right. With increasing concentration of the respective particles, there are differences in the behaviour of the oxidation peak current. It appears that the active charge carriers present in the solution are hindered by the bare CuNPs due to some screening effect or interaction (very high concentration of CuNP reacts with the solution) and lowers the current at electrode. But a protein coating of the CuNP, helps in uninterrupted diffusion of the charge carriers towards electrode. With increase in protein layers maximum numbers of charge carriers reach the electrode increasing the amount of the current.

**Fig. 7:**
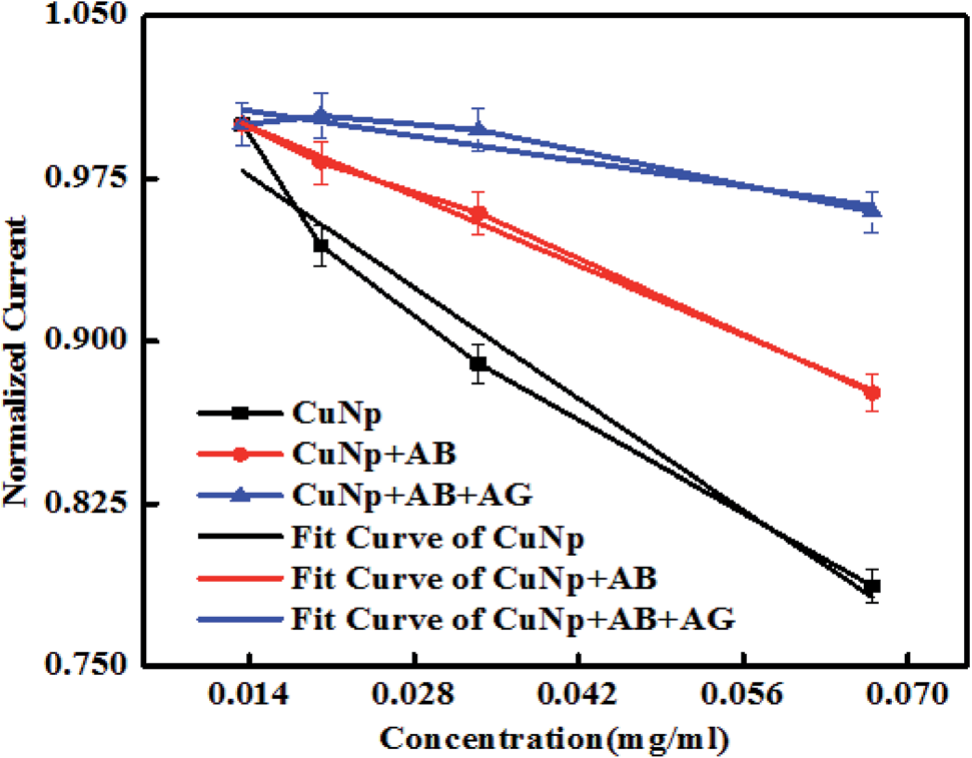
Shows the linear region of the plot (shown in Fig. 6) with variable slopes.

It is also observed that the differences in the behaviour of the oxidation peak current creates a variation in slopes between CuNP, CuNP + AB and CuNP + AB + AG (antibody and antigen). The slopes when calculated for *n* number of measurements (*n* = 2 or more) the values are found to be in the range of (a) CuNP = −3.7637; (b) CuNP conjugated Antibody = −2.3044; (c) CuNP Antibody–GFP = −0.8332 shown in Fig. 7. Therefore, the immune reaction is well separated from the other two and on the basis of the differences in the slopes and the presence of antigen in the solution could be detected.

**Fig. 8:**
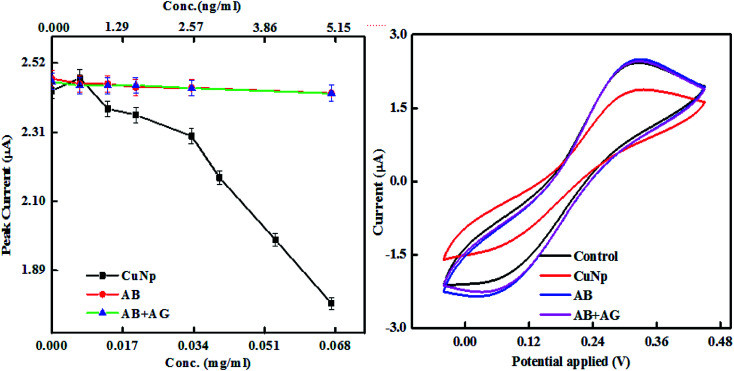
The peak current *vs.* concentration of anti-GFP–antibody and anti-GFP–antibody + GFP immunocomplex (without conjugated to CuNP) is plotted here. The current *vs.* potential curve of the same is also shown together with CuNP and control redox solution at the last point of measurement.

The nature of the graphs in case of nonconjugated immunocomplex (the controls), is shown in Fig. 8. When increase in concentration (*X*-axis) *vs.* the oxidation peak current (*Y*-axis) from nonconjugated antibody (AB) and nonconjugated antibody–antigen reaction complex (AB + AG) is plotted, it was seen that at nanogram level, the immuno reaction between anti-GFP–antibody and GFP cannot be electronically well separated. But as shown earlier (Fig. 6) that on conjugation to CuNP, the same concentration of antibody and GFP complex is detectable. The potential *vs.* current graph also denotes the same (plot on the right panel). All the values from the protein solutions (AB/AB + AG) shows the same potential *vs.* current curve found for the control redox solution (PBS, K_3_FeCN_6_/K_4_FeCN_6_ redox probe and KCl as supporting electrolyte) curve, exhibited at the last point of measurement.

**Fig. 9:**
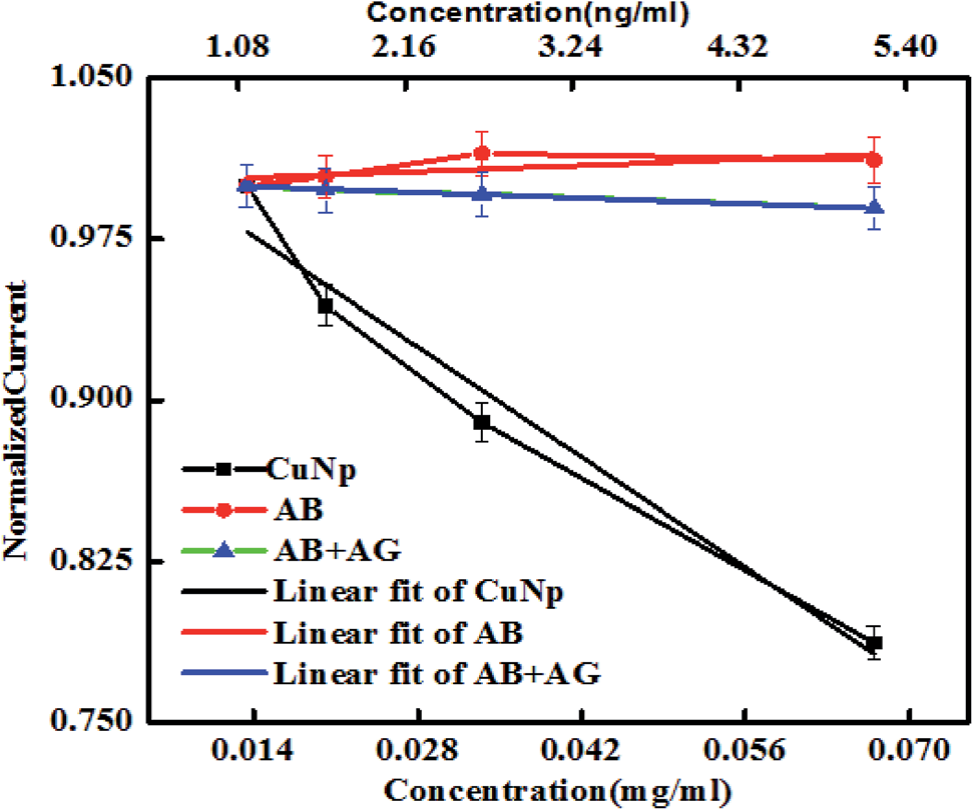
Shows the linear region of the plot (shown in Fig. 8) with fit.

The AB and AB + AG immunocomplex when not conjugated to the CuNP, did not show much differences in the plots unlike conjugated metal nanoparticles in Fig. 6 and cannot be distinguished by eye. The slopes for the anti-GFP–antibody and anti-GFP–antibody + GFP immunocomplex were found to be 0.2 and −0.19 respectively with low Chi square values. Our experiment shows that the results were differentiated well when conjugated to the nanoparticles (Fig. 9).

Therefore, this method could be established as an electronic diagnostic system that rapidly detects antigen (analyte) in solution based on the variation in slopes.

## IV. Conclusion

In conclusion we can say that CuNPs were synthesized by EEW (Electro Explosion of Wire) technique where a Cu wire of 0.5 micron in diameter was exploded on a Cu plate, triggered by high current densities in the wire. After explosion the particles were collected, characterized and bio-functionalized with antibodies. The CuNP, just synthesized in water by EEW were found to be very active and the antibodies were easily conjugated to the particles through surface adsorption technique. Anti GFP antibody was used for conjugation reaction and the immunoreaction with GFP was done to prove that the conjugation was successful. Cyclic voltammetry studies of the immunoreaction (between antibody functionalized CuNP and the respective antigen) was monitored for electronic sensing. The immunoreaction was anticipated to be specific because the newly synthesized bare CuNP could adsorb protein/antibodies only within first 2–3 days of synthesis before they are transformed to oxide. And to avoid adsorption of the GFP (antigen), the electronic sensing was done after this time frame. The concentration (nanogram) at which the immune reaction between antibody and antigen becomes insensitive, the nanoparticle conjugated immunoreaction is well separated. The concentration dependent difference in the slope of the immunocomplex curve against controls reveals whether the immune reaction has taken place or not. It was noted that the metal particles synthesized by this EEW technique can be used for such biosensing reactions. Hence, an electronic diagnostic system that rapidly detects the presence of low concentration of antigen (analyte) based on the variation in slopes could be built. As this electronic measurement is rapid and sensitive, they can replace time-consuming, multistep, label-based assays. So, this method might serve as one of the easiest and fast method for detection of an analyte (antigen), to detect any infectious disease. In addition, this method is not restricted to only antigen–antibody binding but can be used for several other biological interaction studies. For example, the binding studies between two proteins, ligand and receptor or enzyme and substrate can be well monitored.

## Conflicts of interest

An Indian patent has been filed based on this work, Patent Application No. 202011003883 filed in India on 29/01/2020.

## Supplementary Material

RA-010-D0RA07582E-s001

RA-010-D0RA07582E-s002
