# Supplementary material for: A method to detect immunoreactions on the basis of current vs. concentration slope – an electrochemical approach
Source: RSC Adv. 2020 Dec 18;10(73):44798–804. doi: 10.1039/d0ra07582e (PMC9058649; doi:10.1039/d0ra07582e)
Supplement: RA-010-D0RA07582E-s002 [file RA-010-D0RA07582E-s002.pdf]

Electronic Supplementary Material (ESI) for RSC Advances.
This journal is © The Royal Society of Chemistry 2020

Supplementary Material

A method to detect immunoreactions on the basis of current Vs concentration slope – An Electrochemical Approach

Jagriti Gupta[a)], Jyoti Bisht[b)], Mini Agrawal[c)], Jaydeep Bhattacharya[a)], Prasenjit Sen[d)] and Ranjita Ghosh Moulick[c)] *

[a)] School of Biotechnology, Jawaharlal Nehru University, New Delhi 110067
[b)] Department of Physics and Astronomy, NIT Rourkella, Odissa 769008
[c)] Amity Institute of Integrative Sciences and Health, Amity University Haryana, Amity Education Valley, Haryana – 122413
[d)] School of Physical Sciences, Jawaharlal Nehru University, New Delhi, 110067, India

Corresponding author mail: ranjita.ghoshmoulick@gmail.com

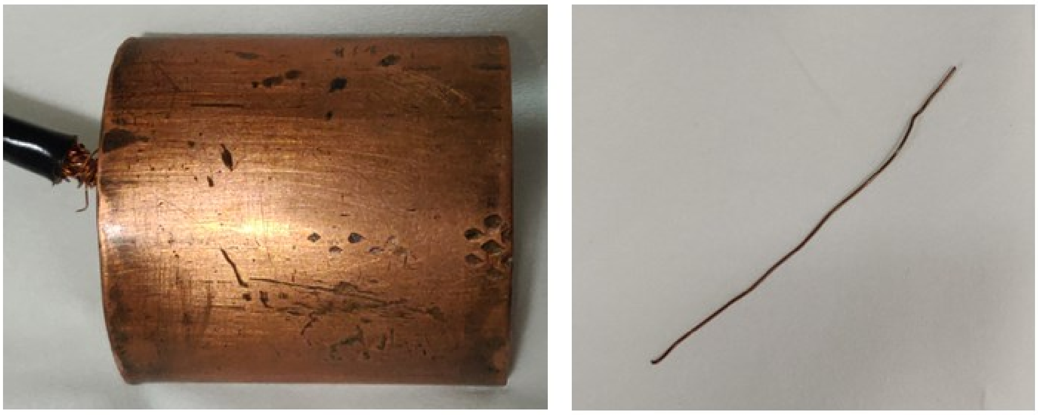

Supplementary Fig 1: Copper plate and copper wire used in the synthesis process

Supplementary Fig. 1 represents the Copper plate and the copper wire from the set up. Both of them were connected to two terminals of the power supply and were brought in contact at regular intervals for explosion to synthesize copper nanoparticles.

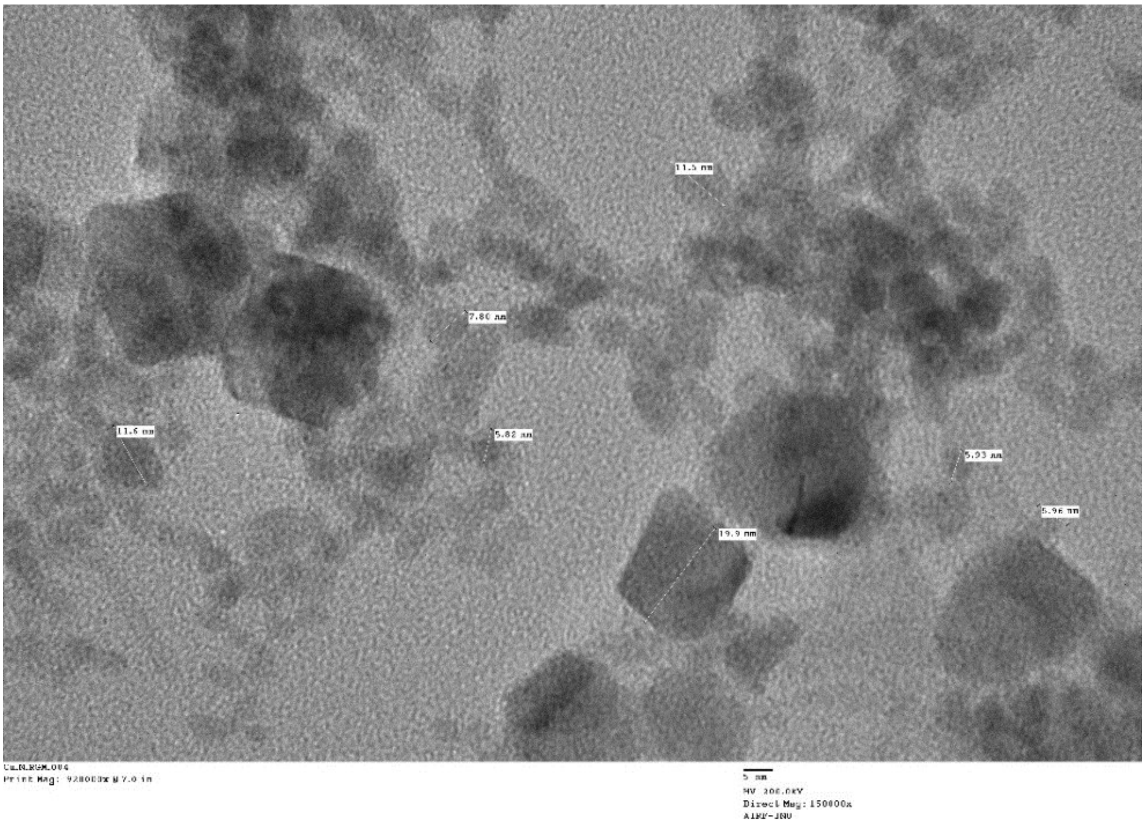

Supplementary Fig. 2 TEM image to show disintegrated particles in the range of 5nm-20nm.

The image (Supplementary Fig. 2) shows presence of CuNP particles in the range of 5-20nm. Though smaller particles in the range of 5-7 nm predominates in the distribution. Due to nonuniform disintegration bigger particles above 10nm is also seen sometimes.

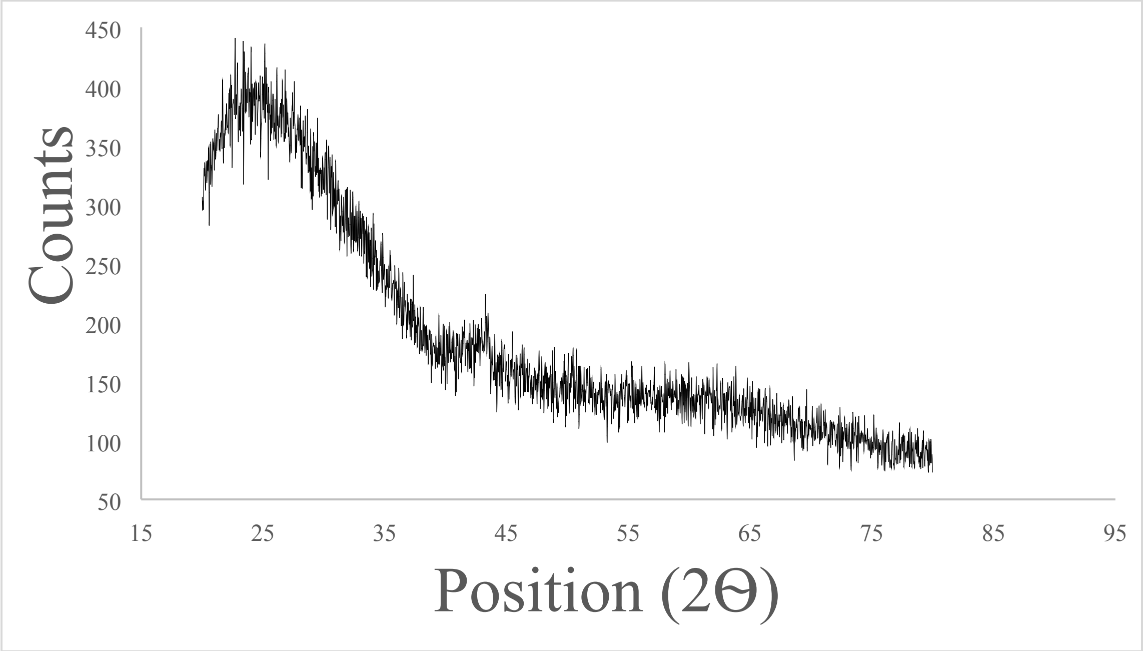

Supplementary Fig. 3 XRD of the CuNP prepared by EEW technique.

Supplementary Fig. 3 represents XRD pattern of CuNP. A small peak at 2θ value of 43.28 corresponds to (111) plane of metallic copper (https://shodhganga.inflibnet.ac.in/bitstream/10603/90129/9/09_chapter3.pdf).

Time (min)	Zeta potential (mV)	Time(min)	Zeta potential (mV)
0	-19.3	30	-19.7
3	-19.5	33	-22.0
6	-19.5	36	-18.9
9	-18.2	39	-18.5
12	-19.0	42	-18.9
15	-19.1	45	-18.5
18	-20.0	47	-18.4
21	-17.7	51	-18.7
24	-19.7	54	-19.5
27	-20.5	57	-19.0

Supplementary Table 1 shows the zeta potential value measured every 3 mins after conjugation reaction (binding of antibody to CuNP) for an hour. A stable zeta value reflects that the adsorption reaction is complete.

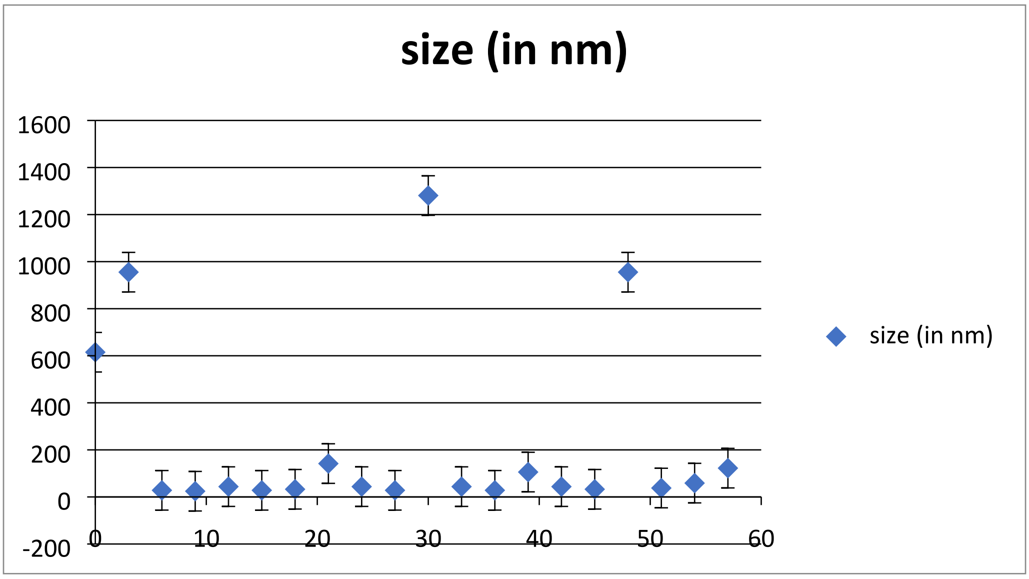

Supplementary Fig. 4 shows the size of anti GFP antibody conjugated CuNP after mixing until an hour.

The above measurements exhibited that before the conjugation process both the CuNP and the ani-GFP-Antibody existed in an aggregated form. But upon binding they form smaller complexes in the range of 24-58nm recorded every 3 mins for an hour. Sometimes much higher clusters are also seen during the measurement but the frequency is quite low.
